# Third dose of the BNT162b2 vaccine in cardiothoracic transplant recipients: predictive factors for humoral response

**DOI:** 10.1007/s00392-022-02075-2

**Published:** 2022-08-22

**Authors:** Angelika Costard-Jäckle, René Schramm, Bastian Fischer, Rasmus Rivinius, Raphael Bruno, Benjamin Müller, Armin Zittermann, Udo Boeken, Ralf Westenfeld, Cornelius Knabbe, Jan Gummert

**Affiliations:** 1 Clinic for Thoracic and Cardiovascular Surgery, Heart and Diabetes Center Northrhine Westfalia, University Hospital, Ruhr-University Bochum, Georgstr. 11, 32545 Bad Oeynhausen, Germany; 2 Institute for Transfusion- and Laboratory Medicine, Heart and Diabetes Center Northrhine Westfalia, University Hospital, Ruhr-University Bochum, Georgstr. 11, 32545 Bad Oeynhausen, Germany; 3 Clinic for Cardiology, Angiology and Pneumology, University Clinic Heidelberg, Im Neuenheimer Feld 672, 69120 Heidelberg, Germany; 4 Clinic for Cardiac Surgery, University Clinic Düsseldorf, Heinrich Heine University Düsseldorf, Moorenstr. 5, 40225 Düsseldorf, Germany

**Keywords:** COVID-19, Heart transplant recipients, BNT 162b2 vaccine, Booster dose, Immunosuppression

## Abstract

**Background:**

We report the results of a prospective study on the immunogenicity of a 3rd dose of BNT162b2 in thoracic organ recipients with no or minimal response following a two-dose BNT162b2 vaccination scheme.

**Methods:**

A total of 243 transplant recipients received a homologue 3rd dose. Anti-SARS-CoV2-immunoglobulins (IgGs) were monitored immediately before (T1), 4 weeks (T2) as well as 2 and 4 months after the 3rd dose. Neutralizing antibody capacity (NAC) was determined at T2. To reveal predictors for detectable humoral response, patients were divided into a positive response group (*n* = 129) based on the combined criteria of IgGs and NAC above the defined cut-offs at T2—and a group with negative response (*n* = 114), with both, IgGs and NAC beyond the cut-offs.

**Results:**

The 3rd dose induced a positive humoral response in 53% of patients at T2, 47% were still non-responsive. Sero-positivity was significantly stronger in patients who presented with weak, but detectable IgGs already prior to the booster (T1), when compared to those with no detectable response at T1. Multivariable analysis identified age > 55 years, a period since transplantation < 2 years, a reduced glomerular filtration rate, a triple immunosuppressive regimen, and the use of tacrolimus and of mycophenolate as independent risk factors for lack of humoral response.

**Conclusions:**

Our data indicate that a lack of immunogenicity is linked to the type and extent of maintenance immunosuppression. The necessity of the cumulative immunosuppressive regimen might individually be questioned and possibly be reduced to enhance the chance of an immune response following an additional booster dose.

## Introduction

Vaccines against coronavirus disease 19 (COVID-19) have been proven to be safe and highly efficient, showing robust immunogenicity and protection against severe disease manifestation [1, 2]. As opposed to the general population, immunocompromised individuals in general and especially solid organ transplant recipients (SOTR) show markedly attenuated responsiveness [3].

We have previously reported a poor humoral and cellular immune response of thoracic organ transplant recipients to a two-dose vaccination with BNT162b2 [4]. This observation was in line with reports of breakthrough infections with severe disease course in fully vaccinated SOTR [5, 6], highlighting the necessity of adapted vaccination protocols for this at-risk population.

An additional booster dose had been proposed, and first reports on mixed cohorts [7–9] and more recently on heart [10] and lung [11] transplant recipients reported varying seroconversion rates between 13% [11] and 67% [10].

Herein, we report our experience with a third BNT162b2 dose, analysing humoral responses by serially measuring the titres of anti-SARS-CoV-2 spike-protein antibodies of immunoglobulin G class (IgG) as well as neutralizing antibody inhibitory capacities (NACs) following the 3rd dose. Additionally, we identified predictors of immunogenicity, with an explicit focus on the type and strength of the maintenance immunosuppressive therapy as a potential suppressor of an appropriate antibody response to the mRNA vaccine.

## Methods

Cardiothoracic transplant recipients who had undergone the two-dose vaccination with the mRNA vaccines BNT162B2 were recruited through their German transplant centres to participate in this prospective single-arm trial. The study had been approved by the Robert-Koch-Institute as Clinical Trial (EudraCT 2021-002,554-90) and by the Institutional review board of the Oeynhausen University Hospital and is compliant with the ISHLT statement on transplant ethics. Participants provided informed consent.

According to the study protocol, a blood sample was drawn for screening to assure that the inclusion criterion of an anti-SARS-CoV-IgG titre < 160 BAU/ml, corresponding to the 5% percentile of the response in a cohort of health care workers [4] was met. A titre < 160 BAU/ml was considered as a weak humoral response to the two-dose vaccination. Application of the 3rd booster dose was possible ≥ 8 weeks after 2nd dose.

Exclusion criteria were transplantation within the last 6 months, acute infections at time of vaccination, a history of prior COVID-19 infection, and rejection episodes within the last 3 months. All patients underwent evaluation on day of vaccination to verify clinical stability. A week post vaccination, a safety (phone) visit was established, in addition, a questionnaire was requested to screen for any adverse events, both for local reactions as well as systemic reactions. Patients were followed 4 months to detect unsolicited adverse events (AE), serious AE or medical attended side effects.

Clinical data were collected from the electronic patient data base. The individual maintenance immunosuppressive regimen was recorded, capturing the daily compound doses as well as the current calcineurin inhibitor (CNI) and/or mammalian target of rapamycin (mTOR) inhibitor trough plasma levels. Laboratory biochemistry parameters included serum creatinine levels, total white blood cell count, absolute lymphocyte count, serum bilirubin levels, and C-reactive protein.

### Laboratory testing

Blood specimens were drawn prior to booster vaccination (T1) and 28 ± 4 (T2), 56 ± 7 (T3) days, and in case of a positive response at T2 also 120 ± 7 days (T4) thereafter. IgG titres were determined at all sampling points. NAC was analysed at T2.

The commercial ELISA SARS-CoV-2 IgG II quant (Abbot, Lake Forrest, USA), a chemiluminescent microparticle immunoassay (CMIA) was used for quantitative determination of IgGs. Data were expressed in WHO-standardised units BAU (binding antibody unit) per ml. According to the manufacturer’s recommendation, values < 7.1 BAU/ml were regarded as negative, values ≥ 7.1 BAU/ml were interpreted as positive. According to the manufacturer, this CMIA displays clinical sensitivity and specificity of 98.81–99.55%, respectively.

To test for circulating neutralizing antibodies, a surrogate virus neutralization test (SVNT) measuring the %-inhibition capability of binding to ACE2-receptors was used (NeutraLISA™ SARS-CoV-2, Euroimmun, Lübeck, Germany).

**Fig. 1:**
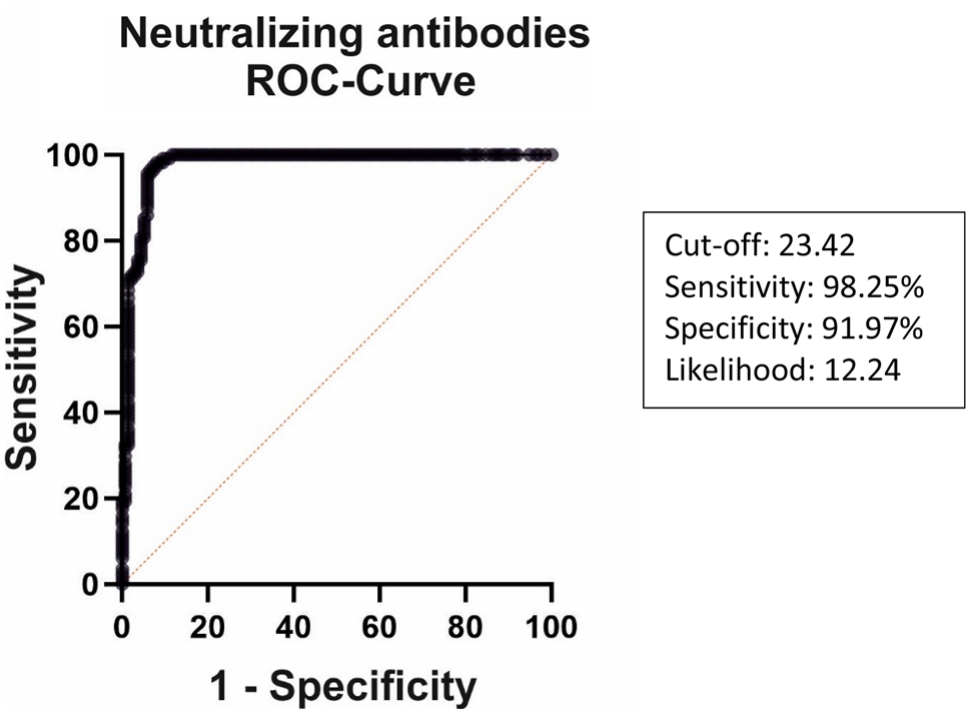
ROC analysis to identify the cut-offs for positive Neutralizing antibody capacity (NAC) based on the results of IgG-binding antibody data obtained 4 weeks after 3rd dose. The resulting cut-off was 23.42%-inhibition (Likelihood ratio: 12.24) with a sensitivity of 98.25% (95% CI 93.83% to 99.69%) and a specificity of 91.97% (95% CI 86.19% to 95.46%). ROC, receiver operating characteristics

A ROC analysis was used to obtain cut-off values for neutralizing antibody capacity (NAC). The analysis was performed based on IgG-binding data obtained at T2 (cut-off ≥ 7.1 BAU/ml classified as responder). The resulting cut-off for NAC was 23.42%-inhibition (Likelihood ratio: 12.24) with a sensitivity of 98.25% (95% CI 93.83–99.69%) and a specificity of 91.97% (95% CI 86.19–95.46%, Fig. 1).

A positive response to the 3rd BNT162b2 dose was defined as the combination of a positive IgG-titre ≥ 7.1 BAU/ml and a neutralizing antibody capacity (NAC) ≥ 23.42% inhibition (IH). Correspondingly, the combination of an IgG-titre < 7.1 BAU/ml, combined with NAC < 23.72% IH was defined as negative response.

### Statistical analysis

Descriptive statistics were expressed as counts and percentages for categorical data, and chi-square test was used to analyze differences between groups. The Mann–Whitney test was used to analyze inter-group differences of numerical data, values were expressed as the median and interquartile range (IQR) or 25th and 75th percentiles).

Univariate and multivariate logistic regression analyses were performed to identify factors associated with a positive antibody response. The variables used in the multivariate analysis were those with a *p* < 0.05 in the univariate analysis. Results are presented as odds ratio (OR), 95% confidence intervals (CIs) and *p*. Statistical significance was set at *p* < 0.05.

The correlation between IgG and log-transformed neutralizing antibodies capacity was analyzed using Spearman´s correlation by two-tailed parametric *t* test means with 95% CI. Data analysis was performed using IBM SPSS Statistics Version 27, Graphics were elaborated using GraphPad Prism9.

## Results

Of the 280 screened patients who had undergone a 2-dose vaccination with BNT 162B2–254 (91%) patients met the inclusion criterion (IgG titre < 160 BAU/ml). 11 patients were excluded from analysis due to discordant humoral responses at T2, with either IgG-titres.

 ≥ 7.1 BAU/ml and NAC < 23.72% IH or IgG < 7.1 BAU/ml and NAC ≥ 23.72% IH, respectively. The resulting study cohort comprised of a total of 243 transplant recipients (228 heart, 14 lung, 1 heart–lung), clearly defined as either sero-positive or sero-negative according to, therefore, mentioned definitions. The median age was 62 (55; 68) years, and 63 (25%) patients were females.

The 3rd dose was given 141 (121; 179) days following the 2nd dose. The median time between transplantation and the initiation of vaccination was 73 (30; 138) months.

Immunosuppressive maintenance therapy included glucocorticoids in 146 (60%) patients, tacrolimus in 172 (71%), mycophenolic acid in 183 (75%), mTOR-inhibitors in 59 (24%) and cyclosporine in 57 patients (23%). Triple immunosuppressive therapy was given in 141 (85%) patients. The combination of tacrolimus-mycophenolic acid and prednisone was most frequently used [84 patients (35%)], including all 15 lung transplant recipients. 97 patients (40%) had been weaned off chronic steroids (Table 1).

**Table 1:** Baseline characteristics of cardiothoracic transplant recipients stratified by antibody response

Parameter	Total cohort *N* = 243	Negative response IgG < 7.1 BAU/ml, NAC < 23,42 *N* = 114	Positive response IgG > 7,1 BAU/ml NAC ≥ 23,42 *N* = 129	*p* value
Age, years^a^	62 (55; 68)	63 (57;68)	60 (53; 68)	0.08
Female sex^b^	63 (25)	35 (31)	28 (22)	0.23
Heart transplantation^b^	228 (94)	99 (87)	129 (100)	< 0.001
Lung/Heart–lung transplantation^b^	15 (6)	15 (13)	0 (0)	< 0.001
Diabetes Mellitus^b^	37 (15)	21 (19)	16 (12)	0.20
Body mass index (kg/m^2^)^a^	25 (22; 28)	25 (22;28)	25 (22; 28)	0.42
eGFR (ml/min/1.73m^2^)^a^	50 (36; 68)^c^	49 (33;63)	56 (37; 72)	0.08
C-Reactive Protein (mg/dL)^a^	0.25 (0.10; 0.52)^c^	0.20 (0.10; 0.57)	0.20 (0.11; 0.45)	0.81
Bilirubin (mg/dL)^a^	0.61 (0.46; 0.87)^c^	0.58 (0.45; 0.76)	0.68 (0.47; 0.94)	0.027
Leucocytes (cells/µL)^a^	6700 (5300; 8400)^c^	6700 (5300; 8300)	7000 (5300; 8600)	0.37
Lymphocytes (cells/µL)^a^	1300 (1000; 1800)^c1^	1150 (800; 1725)	1400 (1100; 1900)	0.15
Time table				
Organ transplantation to 1st vaccine, months^a^	73 (30; 138)	37.9 (21.8;100.0)	99.5 (52.5; 179.7)	< 0.001
Time of 3rd vaccine from 2nd vaccine, days^a^	141 (121;179)	143 (118;181)	141 (126; 176)	0.56
Anti-SARS-CoV-2 IgG at				
Day of 3rd vaccination (T1), BAU/mL^a^	0.8 (0.1;6.7)	0.1 (0.0;0.3)	6.5 (1,7; 19,6)	< 0.001
28 days after 3rd vaccination (T2), BAU/mL^a^	18.3 (0.3;206.7)	0.25 (0.1;1.025)	188.1 (57,8; 590,3)	< 0.001
60 days after 3rd vaccination (T3), BAU/mL	15.4 (0.4;222.4)	0.35 (0.1;0.925)	205,8 (56,9; 664,4)	< 0.001
Neutralizing antibody capacity (NAC)28 days after 3rd vaccination (t2), %	28.2(12.7;84.6)	12.2 (9.3;15.6	82.9 (45,93; 98,84)	< 0.001
Immunosuppression regimen				
Triple therapy^2^	141 (58)	87 (76)	54 (42)	< 0.001
TAC + Mycofenolate + Prednisone^2^	84 (35)	64 (56)	20 (16)	< 0.001
TAC + Mycofenolate^2^	47 (19)	22 (19)	25 (19)	0.42
TAC + mTOR + Prednisone^2^	21 (9)	10 (9)	11 (9)	0.65
TAC + mTOR2	15 (6)	3 (3)	12 (9)	0.040
CSA + Mycofenolate + Prednisone2	21 (9)	8 (7)	13 (10)	0.50
CSA + Mycofenolate2	17 (7)	1 (1)	16 (12)	< 0.001
CSA + EVL + Prednisone2	6 (2)	1 (1)	5 (4)	0.22
CSA + EVL2	6 (2)	–	6 (5)	0.03
mTOR-Inhibitor + Mycofenolate + Prednisone2	2 (1)	1 (1)	1 (1)	> 0.99
mTOR-inhibitor + Mycofenolate2	8 (3)	2 (2)	6 (5)	0.29
Immunosuppression data				
Chronic prednisone2	146 (60)	86 (75)	60 (46)	< 0.001
Prednisone dose, mg/day1	5.0 (2.5; 7.5)	5.0 (4.375; 7.5)	3.75 (2.5; 5.0)	0.001
Mycofenolate Therapy2	183 (75)	99 (87)	84 (65)	< 0.001
Mycophenolate sodium2	159 (65)	88 (77)	71 (55)	< 0.001
Mycophenolate mofetil2	24 (10)	11 (10)	13 (10)	> 0.99
Mycophenolate sodium dose, mg/day1	1500 (1000;2000)	1500 (1250; 2000)	1500 (1000; 2000)	0.96
Mycophenolate sodium dose mg/kg/day	19(14;26)	19.5 (15; 26	19 (13; 27)	0.74
Mycophenolate mofetil dose, mg/day1	720 (360; 1440)	540 (360; 900)	900 (720; 1440)	0.06
Mycofenolate through level, ng/mL1	2.0 (1.4; 2.9)	2.2 (1.5; 3.2)	1.8 (1.2–2.4)	0.007
TAC therapy2	172 (71)	101 (89)	71 (55)	< 0.001
TAC trough level, ng/mL1	6.1 (4.9; 7,9)	7.1 (5.7; 8.6)	5.2 (4.5; 6.2)	< 0.001
CSA therapy2	57 (23)	10 (9)	47 (36)	< 0.001
CSA trough level, ng/mL1	82 (59; 102)	120 (69; 147)	79 (58; 95)	0.006

*IgG* immunoglobulin G, *NAC* neutralizing antibody capacity, *BAU* binding antibody unit, *eGFR* estimated glomerular filtration rate, *TAC* tacrolimus, *mTOR* mammalian target of Rapamycin, *CSA* cyclosporine, *EVL* everolimus

^a^median with 25th and 75th percentiles

^b^number (percentage)

^c^based on 239 patients

^c1^based on 216 patients

**Fig. 2:**
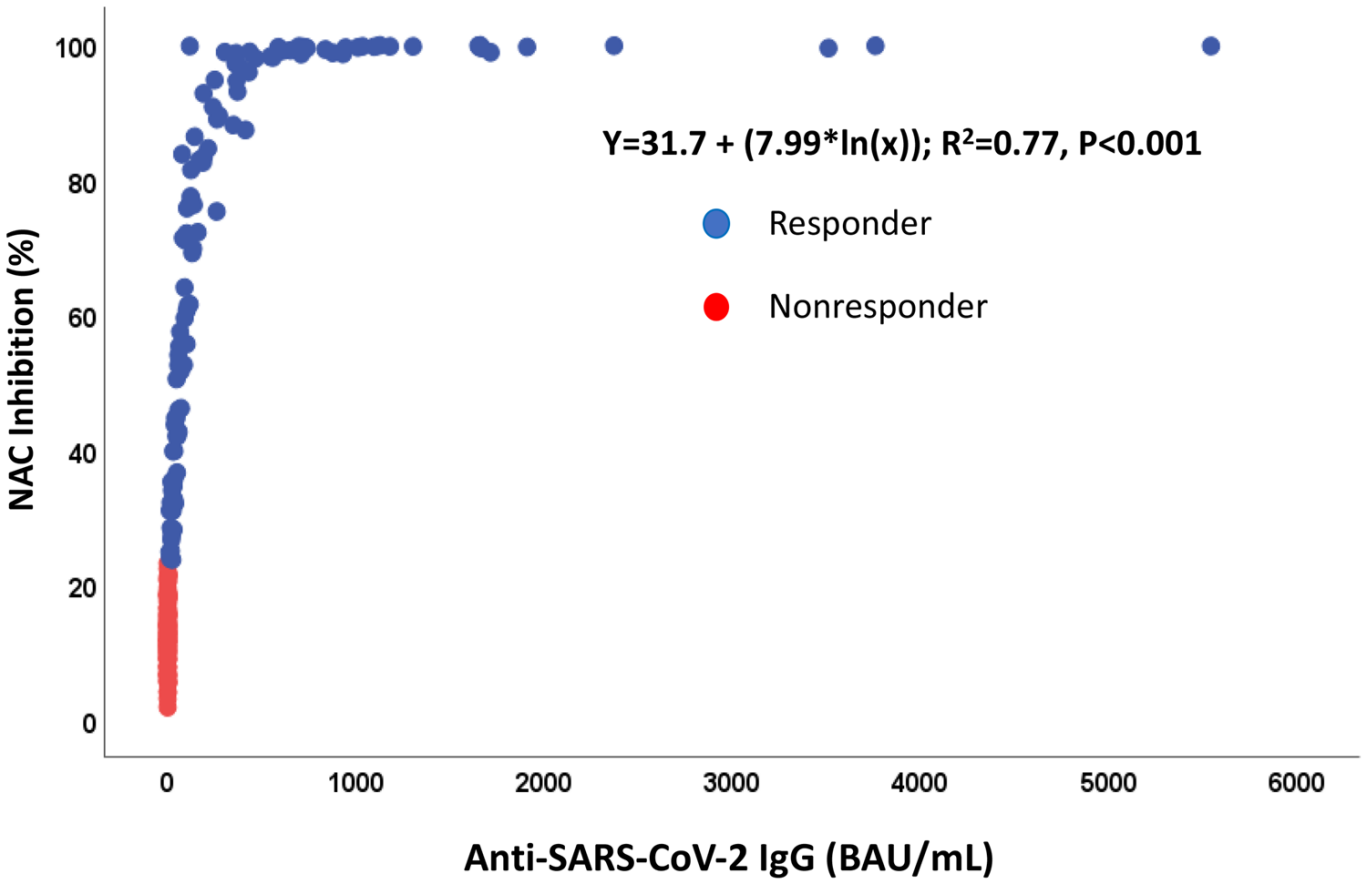
Correlation between anti-SARS-CoV-2 IgG (BAU/ml) and neutralizing antibody capacity (NAC, % inhibition) in cardiothoracic transplant recipients (*n* = 243). Each dot represents a combined IgG and NAC result for 1 participant. Spearman *r* = 0.8426

The median anti-SARS-CoV2-IgG titre for the total cohort at 4 weeks (T2) following the 3rd dose was 18.3(0.3; 206.7) BAU/ml; the median NAC was 28.2 (12.7; 84.6) % IH. We found a significant correlation (*r* = 0.843, *p* < 0.001) between IgG-titres and NACs (Fig. 2).

**Fig. 3:**
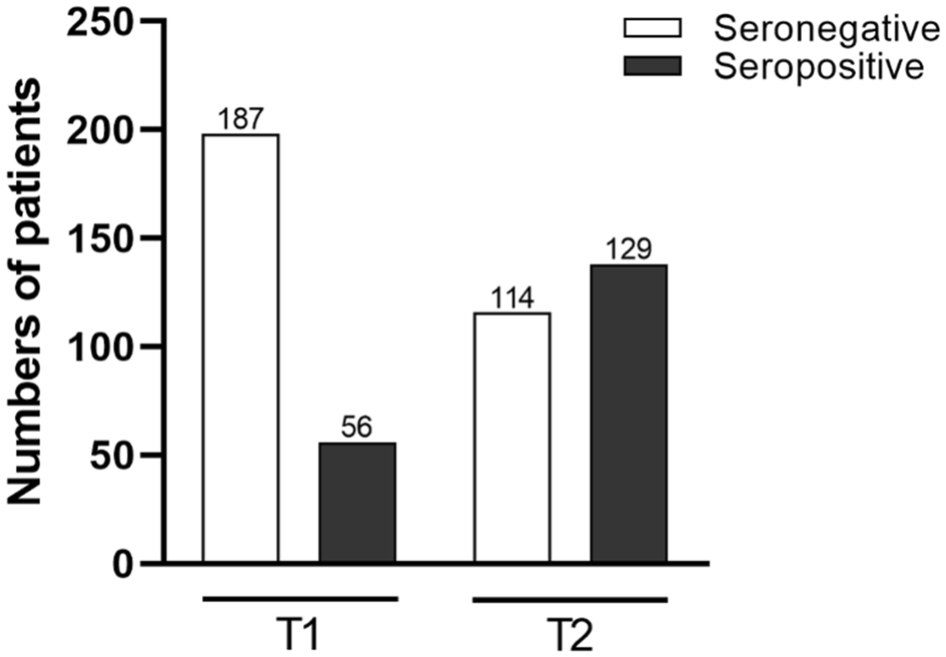
Serostatus before (T1) and 4 weeks after 3rd dose (T2) of BNT162b2 vaccine in 243 cardiothoracic patients

**Fig. 4:**
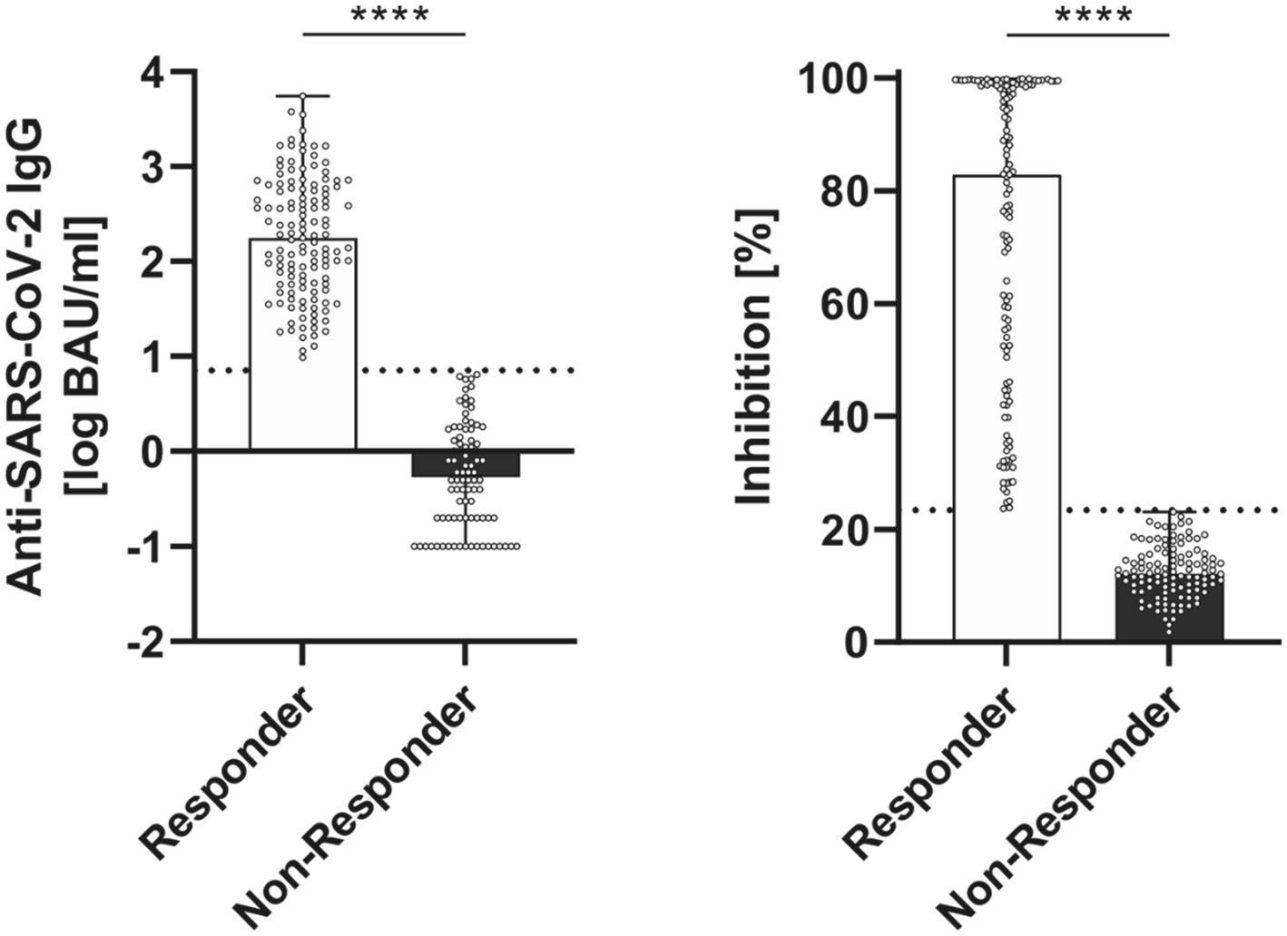
Antibody response to 3^rd^ dose of BNT162b2 vaccine 4 weeks (T2) after booster dose (*n* = 243) **A** Anti-SARS-CoV-2 immunoglobulins (IgG) in BAU/ml **B** Neutralizing antibody capacity (NAC) % inhibition (IH). Antibody-positive cut-offs are indicated by the respective dotted horizontal line. Responders are defined by both, IgG and NAC beyond cut-offs

The defined criterion for sero-positivity (IgGs ≥ 7.1 BAU/ml and NAC ≥ 23.42%) was detected in 129 patients (53%) at T2. The other 114 patients remained sero-negative (Fig. 3). For the sero-positive responders, the median IgG-titre at T2 was 188.1 (57.8; 590.3) BAU/ml with a corresponding median NAC of 82.9 (45.9; 98.9) % inhibition (Fig. 4).

**Fig. 5:**
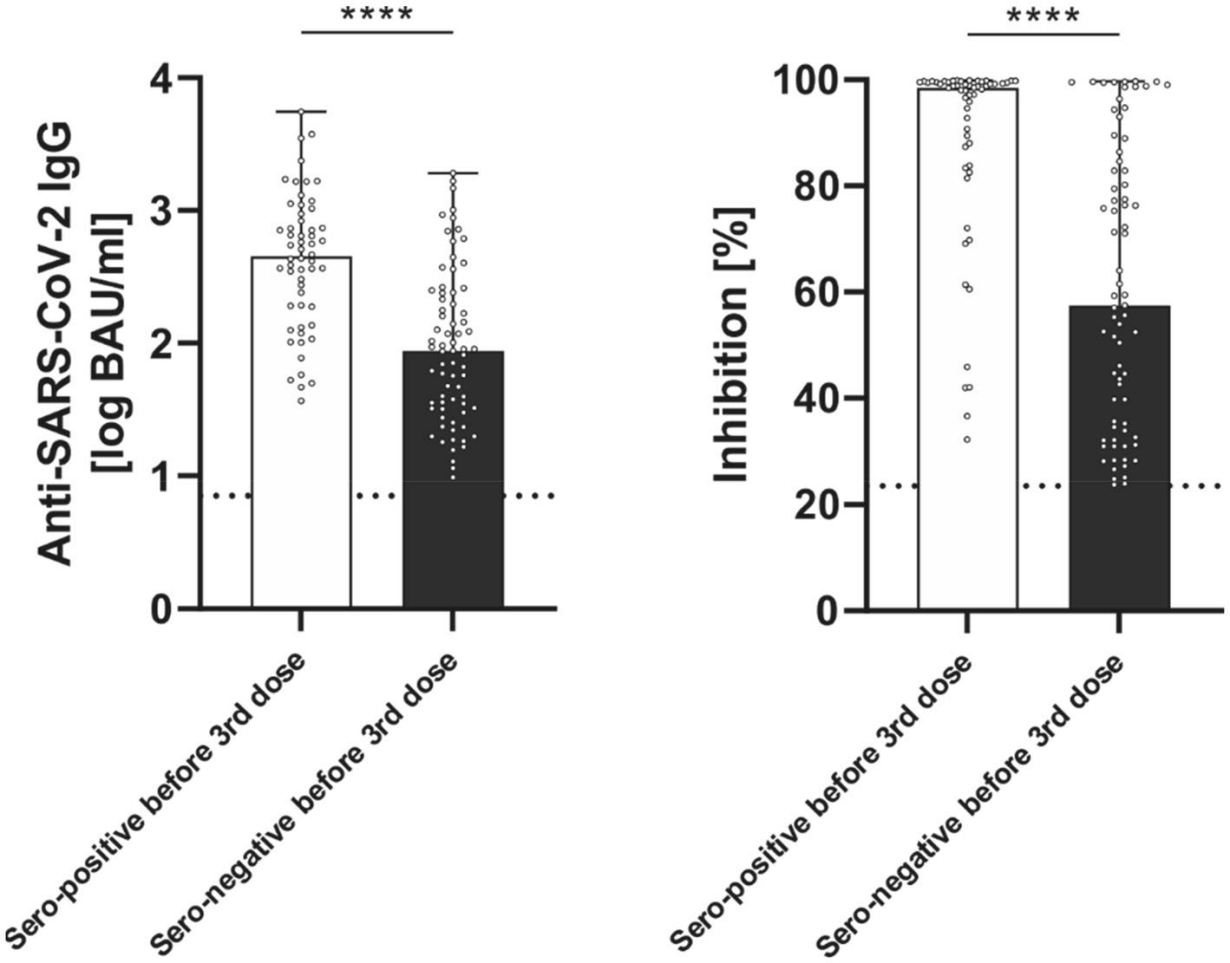
Antibody results of responders to 3rd homologue BNT162b2 dose (*n* = 129) 4 weeks after booster (T2) differentiated according to serostatus before 3rd dose (T1): sero-positive before 3rd dose: > 7, 1, but < 160 BAU/ml; sero-negative before 3rd dose: < 7,1 BAU/ml. **A** Anti-SARS-CoV-2 immunoglobulins (IgG) in BAU/ml; **B** Neutralizing antibody capacity (NAC) % inhibition (IH). Antibody-positive cut-offs are indicated by the dotted horizontal line. Median: red line, 25/75percentiles: black line

Among the 129 sero-positive patients, a fraction of 73 patients had no detectable IgG-titres at T1, prior to the 3rd booster dose. The other 56 patients, sero-positive at T2, had weak (< 160BAU/ml), but detectable (> 7.1 BAU/ml) IgG-titres at T1 (Fig. 3). Notably, the humoral response in sero-positive patients at T2 was significantly more pronounced in the 56 patients with detectable IgG-titres prior to the 3rd dose (T1), when compared to the 73 patients with no detectable IgG-titres at T1 (451 (189; 919) vs. 87 (32;240) BAU/ml, *p* < 0.001, Fig. 5A). Also, NAC was higher at T2 in the subgroup of patients with detectable IgGs at T1 (99(85; 100) vs. 58 (34; 85) % IH; *p* < 0.001, Fig. 5B).

Table 1 presents data on the comparison between patient with and without a humoral response to the 3rd BNT162B2 dose. All 15 lung recipients were non-responders. Non-responders exhibited a shorter median time span between transplantation and the initiation of vaccination compared to sero-positive patients. Non-responders were more frequently on triple immunosuppression, especially on the combination of tacrolimus, mycophenolate, and prednisone. Focussing on individual immunosuppressive compounds, chronic use of prednisone was more common among non-responders as was the use of tacrolimus and of mycophenolate. Conversely, immunosuppression involving mTOR-inhibitors and/or of cyclosporine was more common in responders. Dosage and trough levels of used immunosuppressive drugs did also differ between responders and non-responders: non-responders were treated with higher daily doses of prednisone and presented with higher serum trough levels of tacrolimus.

**Fig. 6:**
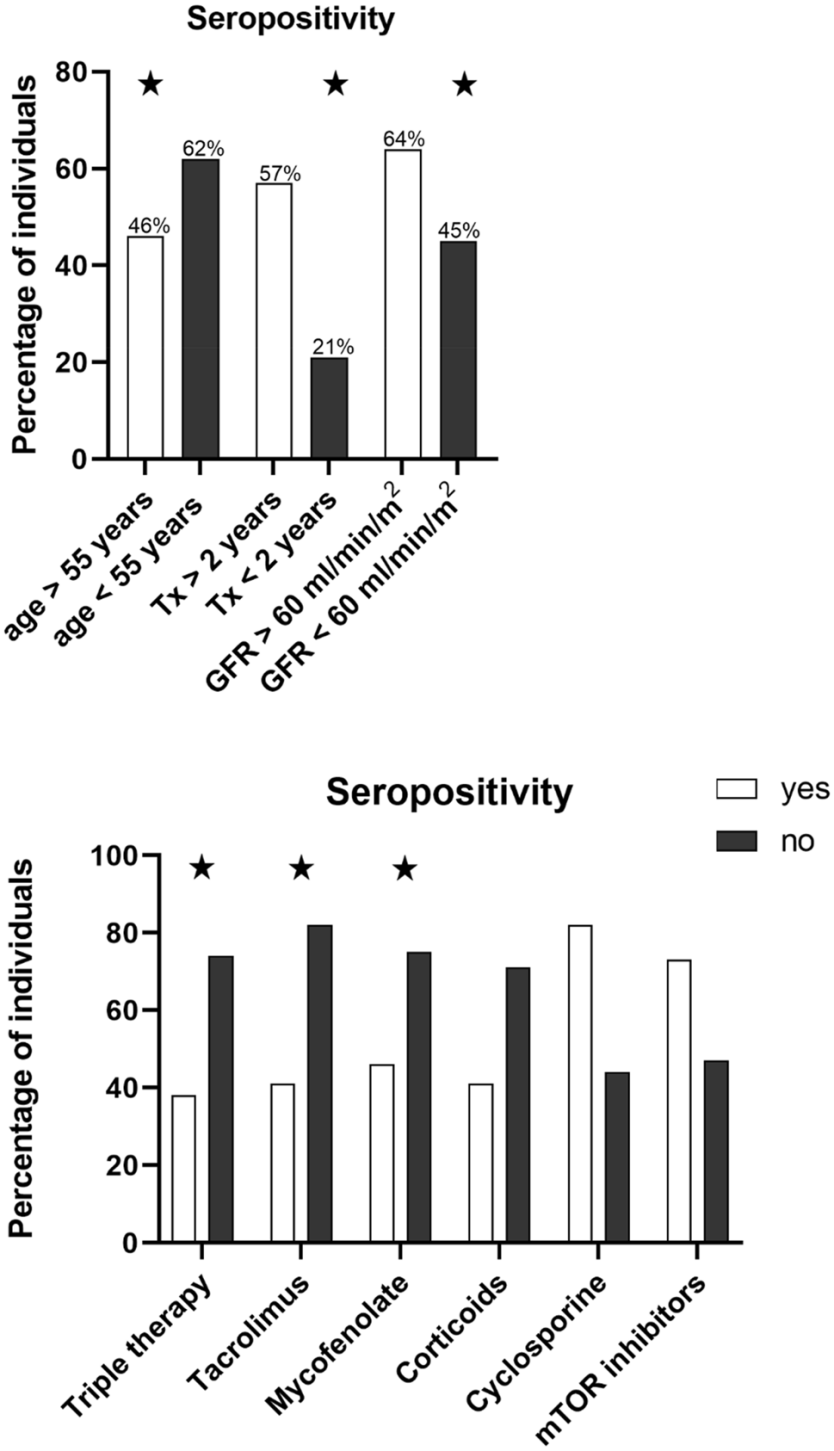
Percentages of cardiothoracic transplant recipients (*n* = 243) with positive humoral response 4 weeks after 3rd homologue BNT162b2 dose, defined as both, anti-SARS-CoV2 IgG and neutralizing antibodies (NAC) beyond cut-offs (IgG ≥ 7.1BAU/ml; NAC ≥ 23.42% Inhibition). ^a^Parameters identified as independent risk factors for lack of positive response by multivariate analysis (Table 2). *Tx* transplantation, *GFR* glomerular filtration rate, *IgG* Immunoglobulins, *IS* immunosuppression, *MMF* mycophenolate, *Tac* tacrolimus

Multivariate analyses identified older recipient age > 55 years, a reduced estimated glomerular filtration rate and a shorter period since transplantation as independent factors associated with a lack of humoral response at T2 (Table 2). In addition, the use of triple immunosuppressive therapy and, regarding individual immunosuppressive compounds, the use of tacrolimus and of mycophenolate were independent predictive factors of sero-negativity at T2 (Fig. 6).

**Table 2:** Logistic regression analysis of independent predictors for lack of humoral response to 3rd BNT162B2 dose

	Univariable OR (95% CI)	*p *value	Multivariate OR (95% CI)	*p* value
Baseline characteristics				
Sex, female	1.60 (0.90; 2.85)			
Age: > 55 years (reference: ≤ 55 years)	1.88 (1.05; 3.35)	.034	3.75 (1.66; 8.48)	.02
BMI	1.00 (0.94; 1.06)			
Diabetes mellitus	1.63 (0.80; 3.30)			
Laboratory data				
Bilirubin (per unit)	0.54 (0.27;1.05)			
C-Reactive Protein (mg/dL)	0.96 (0.73; 1.26)			
Leucocytes (per unit)	0.96 (0.88; 1.05)			
Lymphocytes (per unit)	0.89 (0.61; 1.29)			
eGFR < 60 ml/min/1.73m2 (reference: eGFR ≥ 60 ml/min/1.73m2)	2.41 (1.39; 4.18)	.002	5.08 (2.30; 11.20)	< .001
Time intervals				
Duration between transplantation and vaccination < 2 years	4.85(2.26; 10.39)	< .001	3.38 (1.10; 10.4)	.034
Time of 3rd vaccine from 2nd vaccine, days	1.00 (0.99; 1.00)			
Immunosuppression				
Triple Therapy	4.48 (2.57; 7.80)	< .001	4.00 (1.91; 8.18)	< .001
Tacrolimus therapy	6.35 (3.24; 12.45)	< .001	8.48 (3.65; 19.73)	< .001
Tacrolimus trough level	1.74 (1.40; 2.15)	< .001		
Mycophenolate therapy	3.54 (1.84; 6.79)	< .001	6.10 (2.45; 15.17)	< .001
Mycophenolate daily dose mg/kg	1.00 (1.00; 1.00)			
Chronic Prednisone	3.53 (2.04; 6.12)	< .001		
Prednisone daily dose (mg)	1.28 (1.09; 1.49)	.002		
Cyclosporine therapy	0.17 (0.08; 0.35)	< .001		
Cyclosporin trough level	1.04 (1.01; 1.06)	.006		
Everolimus therapy	0.36 (0.19; 0.68)			
Everolimus trough level	1.13 (0.77; 1.65)			

*BMI* body mass index (kg/m2), CRP, *eGFR* estimated glomerular filtration rate

**Fig. 7:**
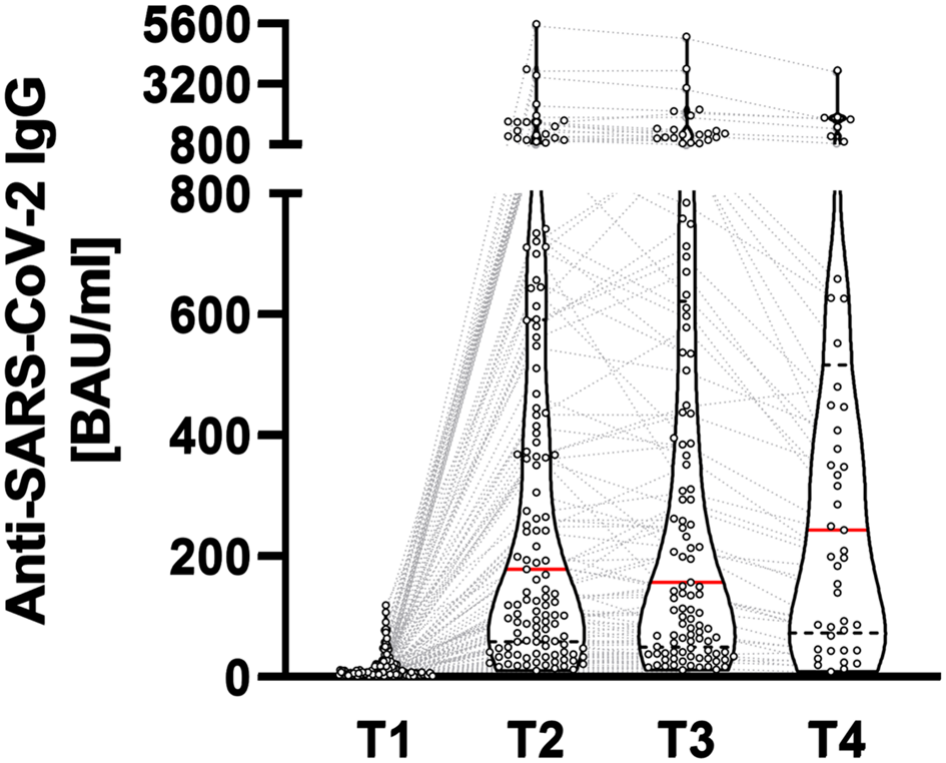
Antibody kinetics of responders (defined by anti-SARS-CoV-2 Immunoglobulins (IgG) and neutralizing antibody capacity beyond cut-offs at T2). T1: before 3rd dose, T2: 4 weeks, T 3: 2 months, T 4: 4 months after 3rd dose. Median: red line, 25/75percentiles: dotted black line

Figure 7 depicts the kinetics of IgG-titres following the booster dose for the patients who were sero-positive at T2. It confirms a durable antibody response with preserved levels of IgG-titres until 4 months (T4) after application of the booster dose. No patient showed a decrease below determination level. The dotted lines illustrate the individual changes. 24 patients showed a further increase of their IgG level at T3 compared to T2.

Adverse events were of mild severity only. Mild transient pain at the injection site was the most frequent solicited local reaction. Within a follow-up period of 4 months, no clinical episode of rejection has occurred, and no patient has developed de-novo synthesis of donor-specific HLA-antibodies.

## Discussion

Immunocompromised individuals have been well recognized to elicit weak responses to a two-dose regimen of the currently available COVID-19 vaccines. Specifically, reports on SOTRs show a high percentage of patients with no immune response, or with antibody titres far below those of healthy cohorts [3, 4, 12, 13]. This poor immunogenicity translates into the report of breakthrough infections in fully vaccinated transplant recipients, [5, 6], with partially severe disease courses being comparable to that of non-vaccinated SOTRs [8, 14].

Immune paresis in SOTRs is not a unique observation for the COVID-19 vaccines. Vaccination strategies directed against other infectious diseases likewise yielded in poor immune responses in SOTRs [15]. Improved seroconversion rates and higher antibody titres have been achieved, for example in studies on influenza vaccination of SOTRs, when higher [16] or additional doses [17] were applied. Together with the reports on fading IgG-titres over time after COVID-19 vaccination even in healthy individuals [18], it was, therefore, logical to offer an additional booster dose, at first to groups at increased risk such as SOTRs with their reported poor response to a two-dose regimen [19].

First reports on the effects of a 3^rd^ dose in SOTRs were encouraging [7, 9], but insights in the responses of cardiothoracic transplant recipients are rare and patient cohorts heterogenous [11, 14].

To the best of our knowledge, the study cohort herein is one of the largest and concise cohorts of cardiothoracic transplant recipients investigated for their humoral response to a 3rd dose of COVID-19 vaccines. Dosing intervals, particularly between 2nd and 3rd dose as well as intervals for blood sampling are homogenous in this well-documented cohort, and missing data are scarce.

Analysing the humoral response to COVID-19 vaccines in SOTRs has frequently been limited to the detection of IgG-titres only, and patients with detectable antibody titres beyond the manufacturers´ threshold levels were suggested to be sero-positive. It remains questionable, whether these in vitro test definitions translate into in vivo immunity against COVID-19. True seroconversion conveying adaptive immunity after vaccination cannot be defined by solely quantitative and non-functional detection of antibody titres. Instead, the neutralizing antibody inhibitory capacity (NAC) has been shown to be highly predictive of protection from symptomatic SARS-COV-2 infection [20, 21]. We, therefore, considered the quantitative detection of both, IgG-titres and NAC, each beyond the predefined cut-off values to at least better approach a definition of sero-positivity following the booster dose.

In line with such previous reports, our present data also show a strong correlation of anti-SARS-CoV-2 IgG titres and NACs. Though our definition of sero-positivity combines a quantitative and qualitative measure of the humoral adaptive immune system, it still remains an open question which antibody titre and/or NAC threshold level may indicate adequate immunity against COVID-19. Obviously, judging adequate immune responses is even more complex, considering that vaccination-induced cellular immunity may not necessarily parallel, but can also be divergent to humoral responses. We could show in a preceding investigation, that T-cell reactivity after basic two-dose vaccination with BNT162b2 was likewise robust as the humoral response in healthy control individuals, but both humoral and cellular responses were virtually absent in a cohort of 50 cardiothoracic transplant recipients [4]. It is a limitation of the present study that the time- and cost-demanding determination of T-cell immunity was not performed.

We found a positive response in 53% of patients, while 47% were still sero-negative after the 3rd dose. Pered and co-workers also analysed the response to a 3rd dose in 96 heart transplant recipients [10], also measuring both IgG-titres and NACs. The somehow discrepant finding of a higher humoral responsiveness of 67% of patients in their study can be explained by the fact that we pre-stratified patients according to their humoral response to the 2nd dose. We offered a 3rd dose exclusively to those with IgG-titers below the 5th percentile of antibody titers in a control cohort of health care workers after the 2nd dose [4].

We noticed a sharp difference in reactogenicity to the 3^rd^ dose between patients without and those with low, but detectable IgG-titers prior to the 3rd booster. While all patients in the latter group showed sero-positivity, with an increase of IgG-titers after the booster, only a fraction of the formerly anergic transplant patients did respond to the booster dose. This finding is in accordance with others, with a reported seroconversion rate in previously negative SOTRs to be as low as 10 to 35%. [7, 9], In contrast, a predictive factor associated with a successful response to a 3rd vaccine dose is the presence, even if minimal, of anti-spike IgG [7, 9] and/or the presence of spike-specific IFN-producing CD4 + cell [23, 24] after the 2nd dose.

The durability of IgG-titers following a two-dose vaccination regimen has been shown to be stable for 6–9 months in the general population [18, 25]. In contrast to the general population, however, SOTRs build up markedly lower antibody titers after vaccination, and the persistence of IgG-titers may follow distinct kinetics and wane more quickly in the immunosuppressed cohort. In accordance with a recent report on a mixed cohort of SOTRs [26], we could demonstrate an overall stability of IgG-titers over a follow-up of 4 month in those patients who mounted at least a moderate positive response to the 3rd booster dose. In some of our sero-positive patients, IgG-titers increased from 4 to 8 weeks post 3rd dose, potentially indicating delayed antibody production in immunosuppressed patients.

In our study, older age and impaired renal function were associated with an impaired humoral response to a 3rd BNT162b2 dose. This is in accordance with other reports involving SOTRs [5, 9, 12, 14, 27]. In general, such data are indicative for an aging immunity with impaired immune reactions in the elderly and multi-morbid patients.

Our data strongly suggest that the cumulative immunosuppressive burden impacts the ability to mount an immune response to the 3rd BNT162b2 dose in cardiothoracic transplant patients: in multivariate analysis, the use of triple immunosuppression was an independent risk factor for a lack of response as was the implementation of tacrolimus and of mycophenolate as single compounds of the individual immunosuppressive regimen. Especially the linkage to the utilization of the antiproliferative agent mycophenolate is in accordance with others [4–7, 10, 11]. Extending these finding the chronic usage of prednisone, as well as higher dosages of prednisone and higher trough levels of the calcineurin-inhibitors were linked to a negative response in our study cohort. Furthermore, we found a shorter time span between transplantation and vaccination to be associated with a lack of response. Again, this is most likely because of the relatively higher net immunosuppression early after transplantation, frequently based on triple therapy involving tacrolimus and mycophenolate in more recent eras.

Acknowledging that the net immunosuppressive burden impacts on the immunogenicity after vaccination against COVID-19, one could speculate whether modulating immunosuppressive regimens may represent a tool to increase the likelihood of a response after another booster dose in so far non-responding patients. Such an approach has been explicitly recommended for persons taking antiproliferative agents for control of autoimmune diseases [28], and a randomized controlled trial will test the safety of immunosuppression reduction when administering a booster dose in stable kidney transplant recipients [29]. In cardiothoracic transplant patients, however, this approach must be handled with greatest care as loss of the allograft during rejection is tantamount with patients’ death. Reducing the maintenance immunosuppression temporarily may be a reasonable option in carefully selected stable patients but requires close follow-up.

Increasing vaccine doses or applications may be safer to improve immunogenicity. The possibility of additional doses of mRNA vaccines has been suggested, especially for patients with an at least weak response following prior doses [22, 30]. Our data support this notion showing that all patients with weak, but detectable anti-SARS-CoC-2-IgG titers prior to the 3rd dose responded at least moderately well. However, there are discouraging results for SOTRs persistently anergic after the 3rd dose after application of a 4th dose [31, 32]. For such patients, passive transfer of anti-SARS-CoV-2 monoclonal antibodies might be the only option [33, 34].

Routine use of serology testing of the immune response to the vaccine is not recommended at present, given the lack of approved cut-offs that can be considered protective against SARS-CoV-2 infection or complicated disease [22]. However, in contrast to the healthy population presenting with a relatively uniform response to the ongoing vaccination strategies, the individual response to the vaccine is not predictable in SOTRs. For this highly vulnerable group, serology testing helps to discriminate patients who are likely to be sufficiently protected from those who lack immunogenicity. For the latter group, it is important to be informed about the individual non-responsiveness and the resulting necessity to maintain non-pharmaceutical protection measures beyond the recommendation for the general population. For the transplant physician, results of testing are mandatory to guide individualized clinical decisions for further modified vaccination strategies.

In conclusion, a 3rd dose of the BNT162b2 vaccine leads to a serological response in a fraction of cardiothoracic transplant recipients, whereas almost half of patients still lack a humoral immune response. Our data demonstrate a strong relation of the immune response with the individual net state of immunosuppressive maintenance therapy. Whether a reduction of immunosuppression around the time of vaccination is justified should individually be balanced against the risk of alloimmune complications and requires further assessment as part of a clinical study.

Our data urge for ongoing vigilance for SOTRs, who are still at risk for breakthrough COVID-19 infections. Given the increased vulnerability to severe COVID-19 disease and the difficulties to induce sufficient immunity against COVID-19, it is highly recommended to complete vaccination before transplantation.

## Data Availability

The data underlying this article will be shared on reasonable request to the corresponding author.
